# Glycochenodeoxycholate Promotes Liver Fibrosis in Mice with Hepatocellular Cholestasis

**DOI:** 10.3390/cells9020281

**Published:** 2020-01-23

**Authors:** Simon Hohenester, Veronika Kanitz, Andreas E. Kremer, Coen C. Paulusma, Ralf Wimmer, Helen Kuehn, Gerald Denk, David Horst, Ronald Oude Elferink, Ulrich Beuers

**Affiliations:** 1 Department of Medicine II, University Hospital, LMU Munich, 81377 Munich, Germany; Ralf.Wimmer@med.uni-muenchen.de (R.W.); Gerald.Denk@med.uni-muenchen.de (G.D.); 2 Institute of Pathology, Faculty of Medicine, LMU Munich, 80337 Munich, Germany; veronika.kanitz@med.unimuenchen.de; 3 Department of Medicine I, Friedrich-Alexander-University Erlangen-Nürnberg, 91054 Erlangen, Germany; andreas.kremer@uk-erlangen.de (A.E.K.); Helen.Kuehn@uk-erlangen.de (H.K.); 4 Tytgat Institute for Liver and Intestinal Research, Department of Gastroenterology and Hepatology, Amsterdam Gastroenterology and Metabolism, Amsterdam UMC, University of Amsterdam, 1018 TV Amsterdam, The Netherlands; c.c.paulusma@amc.uva.nl (C.C.P.); r.p.oude-elferink@amc.uva.nl (R.O.E.); u.h.beuers@amsterdamumc.nl (U.B.); 5 Department of Pathology, Charité—Universitätsmedizin, 10117 Berlin, Germany; david.horst@charite.de

**Keywords:** cholestasis, liver fibrosis, bile salts, hepatic stellate cell, EGFR

## Abstract

Hydrophobic bile salts are considered to promote liver fibrosis in cholestasis. However, evidence for this widely accepted hypothesis remains scarce. In established animal models of cholestasis, e.g., by *Mdr2* knockout, cholestasis and fibrosis are both secondary to biliary damage. Therefore, to test the specific contribution of accumulating bile salts to liver fibrosis in cholestatic disease, we applied the unique model of inducible hepatocellular cholestasis in cholate-fed *Atp8b1^G308V/G308V^* mice. Glycochenodeoxycholate (GCDCA) was supplemented to humanize the murine bile salt pool, as confirmed by HPLC. Biomarkers of cholestasis and liver fibrosis were quantified. Hepatic stellate cells (HSC) isolated from wild-type mice were stimulated with bile salts. Proliferation, cell accumulation, and collagen deposition of HSC were determined. In cholestatic *Atp8b1^G308V/G308V^* mice, increased hepatic expression of αSMA and collagen1a mRNA and excess hepatic collagen deposition indicated development of liver fibrosis only upon GCDCA supplementation. In vitro, numbers of myofibroblasts and deposition of collagen were increased after incubation with hydrophobic but not hydrophilic bile salts, and associated with EGFR and MEK1/2 activation. We concluded that chronic hepatocellular cholestasis alone, independently of biliary damage, induces liver fibrosis in mice in presence of the human bile salt GCDCA. Bile salts may have direct pro-fibrotic effects on HSC, putatively involving EGFR and MEK1/2 signaling.

## 1. Introduction

Cholestatic liver diseases such as primary biliary cholangitis (PBC) or primary sclerosing cholangitis (PSC) are chronic progressive disorders that frequently result in liver cirrhosis, with its subsequent complications. Inborn cholestatic syndromes such as progressive familial intrahepatic cholestasis type 1 (PFIC1) or type 2 (PFIC2), with their underlying dysfunction of a phospholipid flippase (ATP8B1; *ATP8B1*) or the bile salt export pump (BSEP; *ABCB11*), respectively, may also rapidly progress to liver cirrhosis, and frequently necessitate liver transplantation [1]. Despite the different pathogenetic pathways involved in cholestatic disorders, the systemic and hepatic accumulation of hydrophobic bile salts is a shared pathogenic feature [2,3]. In PBC, for example, systemic bile salt levels have been found to be increased up to 20-fold in advanced stages [4]. Current concepts of chronic cholestatic diseases therefore highlight the pro-fibrotic properties of accumulating hydrophobic bile salts in cholestasis. This widely accepted hypothesis was brought forward in the 1970s [2,5] and, to this day, accumulation of hydrophobic bile salts is seen as a driving force of fibrosis in cholestatic liver disease [6,7,8,9].

Accumulating hydrophobic bile salts in cholestasis induce hepatocellular apoptosis and injury [8]. Bile-salt-induced apoptosis involves death receptor-dependent and -independent signaling pathways, as characterized by various research groups [10,11,12,13], including our own [14,15]. Bile-salt-induced hepatocellular apoptosis has mainly been characterized in animal models and in vitro. However, the presence of serum markers of apoptosis in patients with cholestatic liver disease suggests the chronic activation of these pathways in humans [16].

Despite a detailed understanding of the mechanisms of bile-salt-induced liver damage, little is known about the potential pro-fibrotic effects of the accumulating bile salts in cholestasis and underlying mechanisms. A small series of studies have described pro-fibrotic signals in hepatic stellate cells induced by uptake of apoptotic bodies or DNA derived from dying hepatocytes [17,18,19]. Sub-toxic concentrations of bile salts have been found to induce TGFβ signaling from hepatocytes with subsequent HSC activation [20].

Importantly, however, in vivo evidence supporting a pro-fibrotic effect of accumulating bile salts in cholestasis is lacking. Commonly used animal models of cholestasis all represent cholangiocellular cholestasis, i.e., cholestasis induced by biliary damage. In the widely used model of *multidrug resistance protein 2* knockout mice (*Mdr2^−/−^*), both cholestasis and fibrosis occur secondary to biliary damage, inflammation, and sclerosis [21]. This is also the case in inducible models of biliary inflammation such as 3,5-diethoxycarbonyl-1,4-dihydrocollidine (DDC) feeding [22]. Common bile duct ligation also leads to both cholestasis and liver fibrosis, but an elevated biliary pressure, biliary infarction, pronounced parenchymal liver damage, and leakage of bile into sinusoidal blood can all drive liver fibrosis independently of cholestasis [23,24]. The pleiotropic signals caused in all of these models of cholangiocellular cholestasis and biliary damage have made it impossible to discern the contribution of accumulating bile salts to the development of liver fibrosis in vivo. Importantly, available models of hepatocellular cholestasis such as the knockout of the bile salt export pump (BSEP) do not spontaneously develop liver fibrosis [25] and, on the contrary, seem to be protected against cholestatic liver damage in the bile duct ligation model [23] due to adaptive mechanisms. Hence, serious doubts have arisen as to whether there truly is an independent pro-fibrogenic effect of accumulating bile salts in cholestasis [26].

To test the specific contribution of accumulating bile salts to liver fibrosis in cholestasis in vivo, we therefore studied an inducible model of hepatocellular cholestasis independent of bile duct damage. To this aim, we used the *Atp8b1^G308V/G308V^* mouse. Atp8b1 is a phospholipid flippase that is localized in the canalicular membrane of hepatocytes. Atp8b1 maintains the polarity of the outer and inner leaflet of the lipid bilayer and, thus, membrane integrity. In the absence of Atp8b1, cholesterol is extracted from the apical membrane of hepatocytes upon bile salt challenge, e.g., by feeding of cholate (CA) [27]. Depletion of cholesterol from the membrane induces dysfunction of BSEP and subsequent cholestasis [28]. The *Atp8b1^G308V/G308V^* mouse model was developed after G308V/G308V was the first *Atp8b1* mutation identified in humans [29]. Here, we used the established model of CA feeding in *Atp8b1^G308V/G308V^* mice to induce chronic hepatocellular cholestasis.

Studies of cholestasis-induced liver fibrosis in mice may be obfuscated by the highly hydrophilic murine bile salt pool, which is mainly composed of tauromuricholate (TMCA) and taurocholate (TCA) [30], while the predominant bile salt accumulating in human cholestasis is the hydrophobic bile salt glycochenodeoxycholate (GCDCA) [31]. Therefore, we sought to humanize the bile salt pool by adding GCDCA to the diet.

For the first time, we demonstrated the development of liver fibrosis in chronic hepatocellular cholestasis, after humanizing the bile salt pool in the *Atp8b1^G308V/G30^* mouse model by addition of GCDCA. To the best of our knowledge, this is the first in vivo proof of principle of a pro-fibrotic effect of accumulating human hydrophobic bile salts in cholestasis. When elucidating potential molecular mechanisms of the pro-fibrotic properties of human hydrophobic bile salts, we found activation of EGFR-dependent signaling cascades and proliferation and collagen deposition of primary hepatic stellate cells in vitro.

## 2. Materials and Methods

### 2.1. Animal Experiments

All animals received standard care, and the study protocol was in accordance with the institution’s guidelines and approved by local authorities (ROB-55.2Vet-2532.Vet_02-14-193). Results are presented according to the ARRIVE guidelines. *Atp8b1^G308V/G308V^* mice were bred at our institution and C57/BL6 wild-type mice were obtained from Charles River (Sulzfeld, Germany). Male animals were used for in vivo studies at 8 weeks of age. Animals were kept in a 12 h light–dark cycle and housed in an enriched environment with ad libitum access to diet and water. Standard (AIN93G) and experimental diet (AIN93G supplemented with bile salts as indicated in the figure legends) were obtained from ssniff (Soest, Germany).

### 2.2. Serum Biochemistry and Serum Bile Salt Measurements

Serum levels of alkaline phosphatase, bilirubin, and alanine aminotransferase were quantified from fresh serum in a respons^®^ 910 fully automated analyzer (DiaSys, Holzheim, Germany).

Total serum bile salt levels were quantified enzymatically using a Diazyme total bile salts kit (Diazyme Laboratories, Poway, CA, USA) according to the manufacturer’s instructions.

### 2.3. Liver Histology, Immunohistochemistry, and Hydroxyproline Quantification

Paraffin blocks were cut into 4 μm thick slices and mounted on microscope slides (Superfrost plus, Thermo Scientific/Menzel, Braunschweig, Germany). After step-wise deparaffinization and rehydration, slides were stained with hematoxylin and eosin according to standard procedures. Immunohistochemistry was performed against αSMA, using a monoclonal rabbit anti-alpha smooth muscle actin antibody (Abcam, Cambridge, UK). Following antigen retrieval applying ProTaqs V Antigen-Enhancer (Quartett, Berlin, Germany), the primary antibody was incubated for 60 min at room temperature at a dilution of 1:800. Detection was performed using the ImmPRESS anti-rabbit IgG polymer kit (Vector, Burlingame, CA, USA) with the chromogen AEC+ (Agilent Technologies, Santa Clara, CA, USA). Subsequently, counter-staining was done using Gill’s hematoxylin formula (Vector, USA). The presence of αSMA-positive cells was scored by an expert pathologist blinded to the experimental conditions.

Hydroxyproline content was determined according to Edwards et al. [32]. For collagen quantification, slides were stained for 1 h with Direct Red 80 (Sirius Red, Sigma-Aldrich, Darmstadt, Germany) and destained twice in ethanol and once in xylol. Slides were scanned with a Pannoramic Midi Slide Scanner (3DHistech, Budapest, Hungary). The Sirius-Red-positive area was quantified by a blinded operator (RW); from each slide, images of five randomized fields (1 mm^2^) were converted to CIELAB color space, red–green component was thresholded by an automated algorithm, and positive area was measured with ImageJ software (Version 1.51s, NIH, Bethesda, MD, USA).

### 2.4. Hepatic Stellate Cell Isolation and Culture

Isolation of primary mHSC was performed via pronase–collagenase perfusion followed by density gradient centrifugation in 13.2% Nycodenz (Axis-Shield PoC, Oslo, Norway) [33]. Purity of preparation was assessed by confirmation of vitamin A autofluorescence.

Isolated HSCs were allowed to attach for 2 h and were then stimulated with bile salts CDCA, GCDCA, TCDCA, and UDCA (Sigma-Aldrich, Darmstadt, Germany) for the time periods indicated, in the absence or presence of AG1478 (Sigma-Aldrich, Darmstadt, Germany) and PD98059 (Cayman, Ann Arbor, MI, USA). To quantify total DNA as a surrogate of cell number, HSCs were incubated with PicoGreen^®^ (Invitrogen, Carlsbad, CA, USA) and fluorescence signals were detected with a CytoFluor 4000 system (PerSeptive Biosystems, Framingham, MA, USA). Proliferation of HSC was quantified using a BrdU-assay kit (Roche, Penzberg, Germany) according to the manufacturer’s instructions. To quantify total cell count, HSCs, seeded in Lab-Tek II Chamber Slides (Nunc, Rochester, NY, USA), were mounted on cover slides with Vectashield mounting medium including DAPI (Vector, Burlingame, CA, USA). Slides were scanned with a Pannoramic Midi Slide Scanner (3DHistech, Budapest, Hungary) and nucles count was performed with ImageJ2 software on the complete slide (0.7 cm^2^).

### 2.5. Collagen Quantification In Vitro

Cells were washed with PBS and stained for 1 h with 0.1% Sirius Red in saturated picric acid. Cells were then washed three times with 100% ethanol, the bound dye was dissolved in 50% methanol/sodium hydroxide (50 mmol/L), and absorption was measured at 540 nm.

### 2.6. Quantitative Real-Time PCR

Snap-frozen liver tissue was homogenized using a TissueLyser (Qiagen, Hilden, Germany). Subsequently, RNA was isolated according to manufacturer’s protocol (TriZOL, Thermo Fisher Scientific, Waltham, MS, USA). Complementary DNA was synthesized using oligo-dT primer and SCRIPT cDNA Synthesis Kit (Jena Bioscience, Jena, Germany). Quantitative real-time PCR was performed using SensiFast™ Sybr^®^ No-ROX Kit (Bioline, London, UK) in a CFX Connect qPCR System (Bio-Rad Laboratories, Hercules, CA, USA). Primer sequences can be provided on request.

### 2.7. Western Blotting

Proteins were loaded in equal amounts, separated by SDS-PAGE, and transferred onto PVDF membranes (Merck-Millipore, Darmstadt, Germany). Membranes were incubated with polyclonal antibodies against anti-MEK/anti-pMEK, anti-Erk/ anti-pErk, anti-PCNA (all Cell Signaling Technology, Danvers, MS, USA), β-actin or monoclonal antibody against α-smooth muscle actin (SMA) (both Sigma-Aldrich, Darmstadt, Germany), followed by goat-anti-mouse-IgG-HRP antibody (Bio-Rad, Feldkirchen, Germany). Visualization was performed with Clarity™ Western ECL Substrate (Bio-Rad, Feldkirchen, Germany), detected with the ChemoCam (INTAS, Göttingen, Germany).

### 2.8. Statistics

Statistical evaluation was performed using SPSS software (Version 25, IBM, Armonk, NY, USA). Normal distribution of data was tested by Kolmogorov–Smirnov and Shapiro–Wilk test. Subsequently, *t*-test or ANOVA was performed with appropriate post-hoc tests (Fisher’s LSD or Tukey’s), where appropriate. For nonparametric data, Mann–Whitney U test, Kruskall–Wallis, or Pearson’s chi-squared test were performed as appropriate.

## 3. Results

### 3.1. Bile Salt Feeding Induces Chronic Cholestasis in Atp8b1^G308V/G308V^ Mice without Relevant Liver Damage

**Figure 1:**
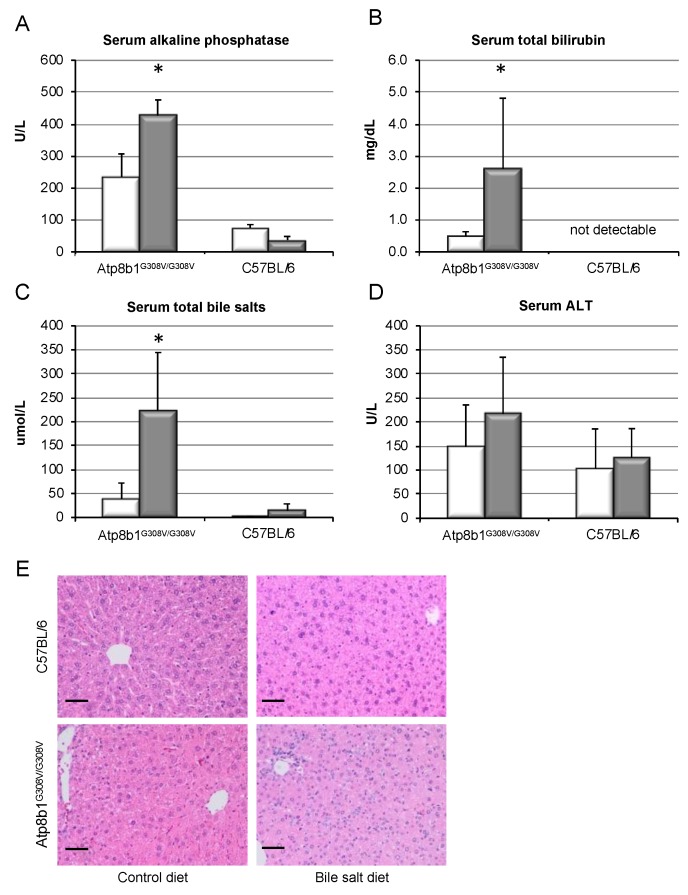
Bile salt feeding induces chronic cholestasis in *Atp8b1^G308V/G308V^* mice. 8 week old *Atp8b1^G308V/G308V^* and wild-type mice (C57BL/6) were fed a standard diet (white bars) or a cholate- (CA, 0.1% *w*/*w*) and glycochenodeoxycholate (GCDCA, 0.3% *w*/*w*)-enriched diet (grey bars) to induce cholestasis and a humanized bile salt pool for 8 weeks. Serum values for alkaline phosphatase (**A**), total bilirubin (**B**), total bile salts (**C**), and ALT (**D**) were determined as described. Results are shown as mean ± standard deviation (*n* = 4 for C57BL/6 and *n* = 7 for *Atp8b1^G308V/G308V^*, * *p* < 0.05, *t*-test). HE stainings were evaluated for liver cell damage, necrosis, or inflammation, without major findings (**E**). All animals showed a non-specific heterogenicity in cell and nuclear size, but not wild-type animals on control diet. Black bar represents 50 µm.

*Atp8b1^G308V/G308V^* mice were fed a cholate (CA)-containing diet (0.1% *w*/*w*) to induce hepatocellular cholestasis as described previously [27,28,34,35]. In order to achieve “humanization” of the murine bile salt pool, glycochenodeoxycholate (GCDCA) was also supplemented in a pre-established concentration (0.3% *w*/*w*) [36]. Feeding was maintained for 8 weeks. Chronic cholestasis was evidenced in *Atp8b1^G308V/G308V^* mice by marked increases in alkaline phosphatase, bilirubin, and total bile salt levels in serum by 1.8-, 5.2, and 5.6-fold, respectively, while wild-type animals remained unaffected (Figure 1A–C). Importantly, supplementation of GCDCA to the diet in absence of CA was ineffective to induce cholestasis in *Atp8b1^G308V/G308V^* mice (Figure S1A).

Despite pronounced cholestasis, serum ALT remained largely unchanged (Figure 1D), indicating an absence of relevant liver cell injury. Since established models of cholestatic liver disease are associated with pronounced liver damage, including significant elevations in ALT, we performed comparative experiments, utilizing *Mdr2^−/−^* mice and DDC feeding. In those models, cholestasis and liver fibrosis were associated with marked (4.1- and 18.8-fold, respectively) increases in ALT levels (Figure S2). In contrast, human chronic cholestatic liver diseases such as PBC are associated with minor elevations of ALT, as exemplified in a recent clinical trial cohort of PBC patients with insufficiently controlled disease, where mean baseline ALT was only marginally elevated (56 U/L) [37].

Upon H&E staining, all animals showed a non-specific heterogenicity in cell and nuclear size (Figure 1E) that was not evident in the wild-type animals on control diet. However, no evidence of hepatocellular damage or necrosis was detected, and no inflammatory infiltrate could be identified in any condition.

Thus, oral intake of CA and GCDCA was able to induce chronic cholestasis in *Atp8b1^G308V/G308V^* mice without detectable liver cell damage or inflammation.

### 3.2. GCDCA Feeding Induces a “Humanized” Bile Salt Pool

As indicated previously [30], GCDCA supplementation in wild-type mice, despite the presumably pronounced rehydroxylation capacity in rodents, leads to a rise in GCDCA plasma levels. While GCDCA was almost undetectable in control mice, GCDCA feeding resulted in an abundance of GCDCA in bile, plasma, and liver of 15.3%, 18.7%, and 16.3% of total bile salts, respectively (Figure S3). Thus, the bile salt pool of mice, which is usually highly hydrophilic and presumably non-toxic, can be shifted towards a more toxic, hydrophobic bile salt composition, resembling a “humanized” bile salt pool.

### 3.3. Chronic Cholestasis, upon Supplementation of GCDCA, Induces Liver Fibrosis in Atp8b1^G308V/G308V^ Mice

**Figure 2:**
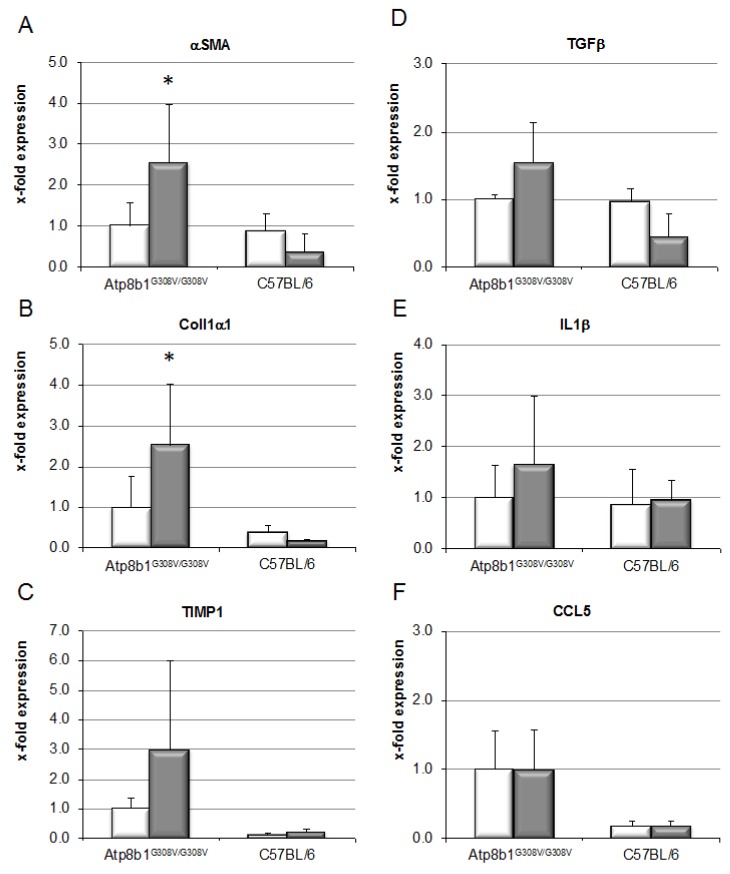
Chronic cholestasis in *Atp8b1^G308V/G308V^* mice leads to pro-fibrotic gene expression only in the presence of GCDCA. *Atp8b1^G308V/G308V^* and wild-type mice (C57BL/6) were fed a standard diet (white bars) or a CA (0.1% *w*/*w*)- plus GCDCA (0.3% *w*/*w*)-enriched diet (grey bars) to induce cholestasis and a humanized bile salt pool for 8 weeks. Quantitative RT-PCR was performed for the indicated genes relative to the house-keeping gene GAPDH. Normalized values are shown as mean ± standard deviation (*n* = 4 for C57BL/6 and *n* = 7 for *Atp8b1^G308V/G308V^*, * *p* < 0.05, Kruskall–Wallis test).

Chronic cholestasis in *Atp8b1^G308V/G308V^* mice induced by feeding of CA, with additional supplementation of GCDCA, for 8 weeks resulted in induction of pro-fibrotic signals, as evidenced by a 2.6- and 2.5-fold upregulation of alpha smooth muscle antigen (αSMA) and collagen 1 alpha (Col1α) mRNA expression over non-cholestatic animals (Figure 2). In line with only marginally elevated serum ALT levels, mRNA levels of inflammatory markers such as IL-1β were not affected (Figure 2).

**Figure 3:**
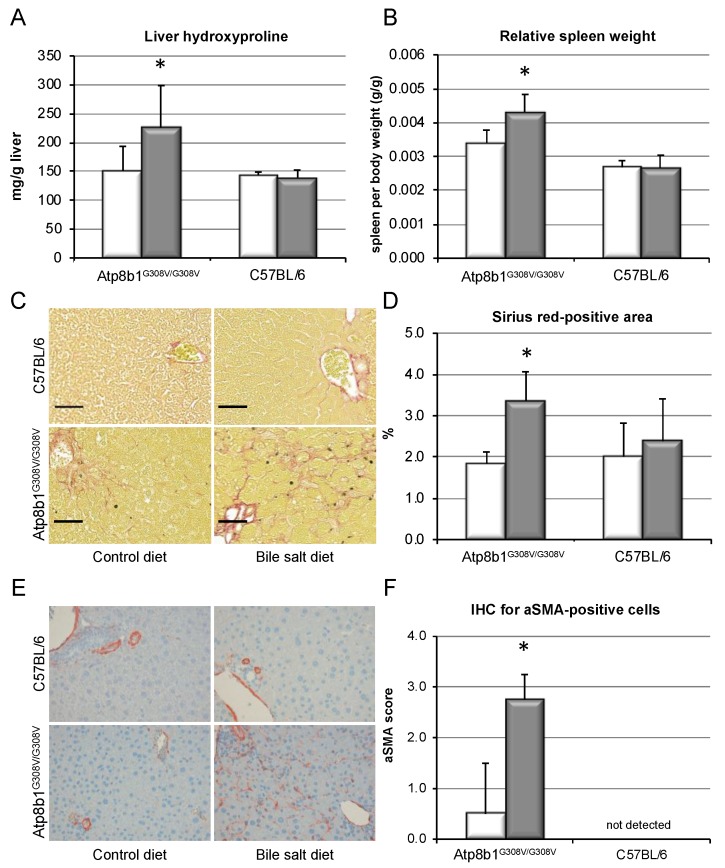
Chronic cholestasis in *Atp8b1^G308V/G308V^* mice induces liver fibrosis in the presence of GCDCA. *Atp8b1^G308V/G308V^* and wild-type mice (C57BL/6) were fed a standard diet (white bars) or a CA (0.1% *w*/*w*)- plus GCDCA (0.3% *w*/*w*)-enriched diet (grey bars) to induce cholestasis and a humanized bile salt pool for 8 weeks. Liver hydroxyproline was determined as described (**A**). Spleen weight was determined at sacrifice and is presented relative to body weight (**B**). Representative Sirius Red staining of liver tissue is shown (**C**, black bar represents 50 µm) and Sirius-Red-positive area was quantified (**D**). Results are shown as mean ± standard deviation (*n* = 4 for C57BL/6 and *n* = 7 for *Atp8b1^G308V/G308V^*, * *p* < 0.05, *t*-test). IHC for αSMA was performed and representative slides are shown (**E**). Blinded histological scoring for αSMA was performed (F) and is expressed as mean ± standard deviation (*n* = 4, * *p* < 0.05, Pearson’s chi-squared test).

Pro-fibrotic signaling in *Atp8b1^G308V/G308V^* mice resulted in increased deposition of collagen in the liver, as demonstrated by an increase in hydroxyproline content from 151.1 ± 42.0 to 225.9 ± 72.9 mg/g liver (*p* < 0.05, Figure 3A). Spleen size was relatively elevated (0.43 ± 0.05 vs. 0.34 ± 0.04%, *p* < 0.05, Figure 3B), suggestive of increased portal pressure secondary to fibrotic changes in the liver, while relative liver weight was unchanged (not shown). Sirius Red staining revealed a reticular distribution pattern of collagen fibers within the livers, with an increase in Sirius-Red-positive area from 1.8 ± 0.8 to 3.4 ± 1.0 % (*p* < 0.05, Figure 3C,D), but no portal–portal bridging fibrosis. IHC for αSMA was performed in order to detect activated HSCs (Figure 3E), and blinded quantification showed a marked accumulation of αSMA-positive cells (Figure 3F). By contrast, non-cholestatic, wild-type animals remained unaffected by bile salt feeding and did not show any signs of liver fibrosis.

It has been speculated that mice may be partly protected from cholestatic liver fibrosis due to their more hydrophilic, non-toxic bile salt pool as compared to humans. In line with this view, cholate feeding alone, without additional GCDCA, did not result in liver fibrosis. Despite induction of marked cholestasis, as depicted in Supplementary Figure S1A,B, levels of hydroxyproline were unaltered (Figure S1C).

In summary, chronic cholestasis led to pro-fibrotic signaling, accumulation of activated HSCs and deposition of excess collagen in mouse livers upon supplementation of the human hydrophobic bile salt GCDCA in vivo, resulting in liver fibrosis. Bile salt feeding alone in the absence of cholestasis i.e., in wild-type mice, however, had no effect on fibrotic signaling. Cholestasis alone, in the absence of GCDCA, also did not result in liver fibrosis in *Atp8b1^G308V/G308V^* mice.

### 3.4. Hydrophobic, but Not Hydrophilic, Bile Salts Promote Proliferation and Collagen Deposition by Primary HSCs

**Figure 4:**
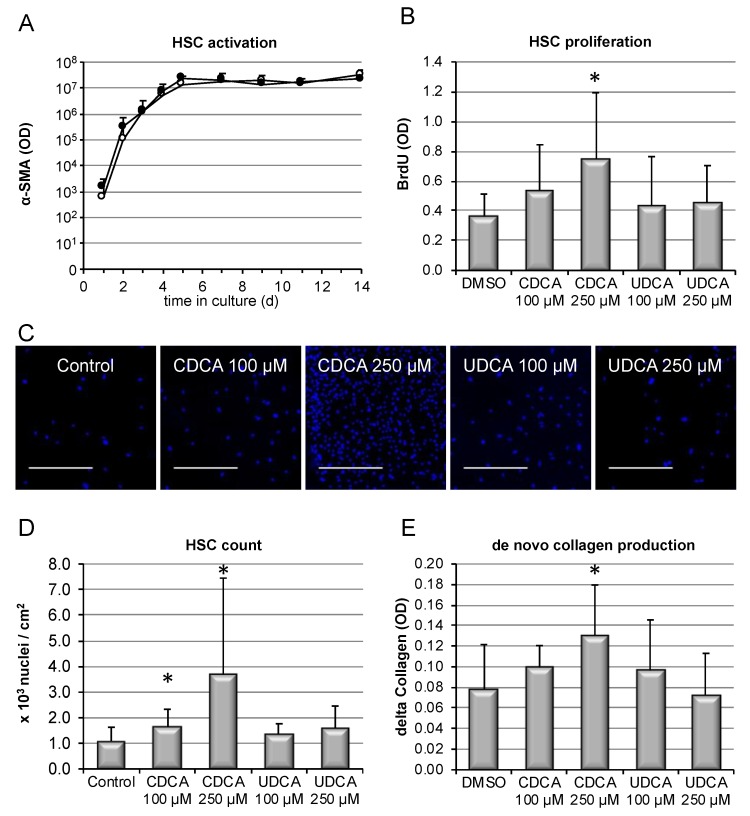
Hydrophobic bile salts promote HSC proliferation and accumulation and collagen deposition. HSCs were isolated from wild-type mice as described above. (**A**) αSMA expression was determined by western blotting during long-term stimulation with CDCA 100 μM (closed circles) or DMSO only (control, open circles) to determine the slope of HSC activation (*n* = 5, each). For subsequent experiments, cells were stimulated with CDCA or UDCA at the indicated concentrations. DNA incorporation was determined after 7 days of culture (**B**). Following a total culture period of 14 days, the absolute number of HSC was determined; cells were seeded in chamber slides, stimulated with the indicated bile salts, stained with DAPI, and pictures were obtained with a slide scanner. Sections of representative pictures are shown in (**C**), where the scale bar represents 500 μm. Resulting cell count was quantified with ImageJ2 software (**D**). To test the functional consequence of excess cell accumulation, de novo collagen production from 7 to 14 days of culture was determined (**E**). Results are shown as mean ± standard deviation (*n* = 8 for B–D, * *p* < 0.05, ANOVA, post-hoc LSD).

To test the hypothesis that bile salts may exert direct pro-fibrotic effects on hepatic stellate cells (HSC), isolated primary HSC were incubated with bile salts in vitro. An established means to determine HSC activation in vitro is the expression of αSMA. Therefore, αSMA expression was monitored for 1–14 days after isolation in presence or absence of CDCA, and the slope of activation was compared to spontaneous activation (control stimulation). As shown in Figure 4A, αSMA protein expression and the slope of HSC activation/differentiation were unaffected by CDCA. Next, HSC proliferation was determined via DNA incorporation assays. Stimulation with CDCA dose-dependently resulted in enhanced proliferation of HSC, while the hydrophilic bile salt UDCA was without effect (Figure 4B). Pro-proliferative signaling was confirmed by detection of PCNA expression on western blotting (Figure S4A). Increased proliferation induced by CDCA, over the course of time, resulted in increased fibroblast numbers, as evidenced by total cell count at the end of the experiments at 14 days of culture (Figure 4C,D), while UDCA was without effect, as was CA (not shown). In consequence, de novo collagen deposition in cell culture was augmented in presence of 250 μM CDCA (Figure 4E).

Thus, the human hydrophobic bile salt CDCA, but not more hydrophilic bile salts, promoted proliferation and aggregation of activated HSC, with subsequently enhanced collagen deposition.

### 3.5. Bile-Salt-Induced HSC Proliferation is Associated with Activation of the MEK/Erk Signaling Cascade

**Figure 5:**
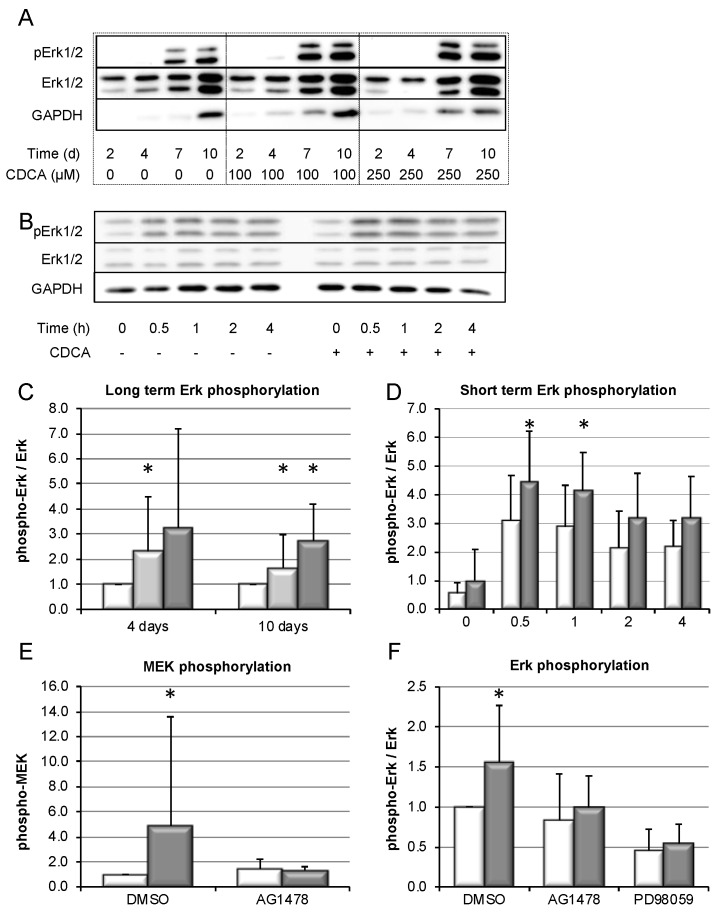
CDCA induces activation of EGFR-, MEK-, and Erk signaling in murine HSCs. HSC were isolated from wild-type mice and stimulated with CDCA for up to 10 days. Activation of Erk was determined by western blotting for phospho-Erk. Representative blots are shown for long-term stimulation with indicated CDCA concentrations (**A**). GAPDH confirmed equal loading for each experimental condition. However, GAPDH expression expectedly changed over time, as HSCs undergo metamorphosis to myofibroblasts during culture, associated with marked metabolic changes. Therefore, total Erk was additionally determined and results are quantified as phospho-Erk/total-Erk. Short-term stimulation with 250 µm CDCA is shown in (**B**), again using total Erk for quantification and providing GAPDH as loading control. Quantification of the results is shown in (**C**) and (**D**) for 100 µm CDCA (light grey bars), 250 µM CDCA (dark grey bars), or DMSO as control (white bars). Furthermore, HSCs were stimulated with CDCA (100 µM, grey bars) compared to control (white bars) for 2–4 h, and inhibitors of EGFR (AG1478, 10 µM) and MEK (PD98059, 50 µM) were added as indicated and compared to control (DMSO). Phosphorylation of MEK (**E**) and Erk (**F**) was quantified and normalized to control. Results are shown as mean ± standard deviation (*n* = 6–8, * *p* < 0.05 compared to control, Mann–Whitney U test).

In order to identify the signaling pathways involved in bile salt-induced proliferation of HSC, western blotting was performed on cell lysates of cultured HSCs. Following stimulation with CDCA for up to 10 days, dose-dependent Erk phosphorylation was detected, which was preserved in long-term culture (Figure 5A,C). Activation of Erk was confirmed in short-term stimulation (Figure 5B,D). Since Erk is a sensitive integrator of various stress signals, some Erk activation was also seen in control cells due to handling for stimulation. However, addition of CDCA to medium induced a significant surplus in Erk phosphorylation. Upstream of Erk activation, CDCA-induced MEK phosphorylation was found (Figure 5E). MEK phosphorylation was abolished by an inhibitor of EGRF signaling, AG1478. Suggesting engagement of an EGFR/MEK dependent signaling pathway in CDCA-induced activation of Erk, phosphorylation of Erk was abolished by both AG1478 as well as PD98059, an inhibitor of MEK activity (Figure 5F).

In summary, CDCA seems to induce an EGFR/MEK-dependent signaling cascade resulting in the activation of Erk. Furthermore, Erk activation was maintained during long-term culture and stimulation of HSCs.

### 3.6. Bile-Salt-Induced HSC Proliferation and Collagen Deposition Is Diminished by MEK/Erk Inhibition

**Figure 6:**
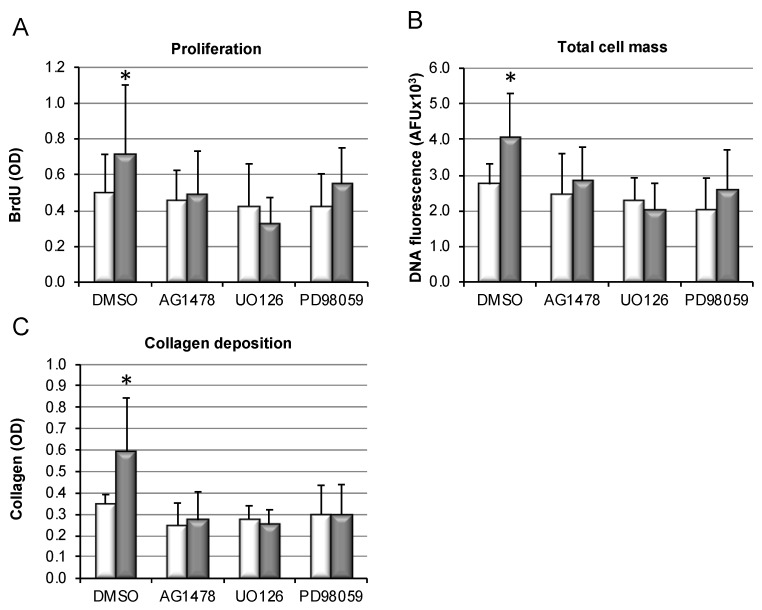
Inhibition of the EGFR/MEK signaling pathway prevents bile-salt-induced proliferation, cell accumulation, and collagen deposition by HSC. HSC were isolated from wildtype mice and stimulated with 250 µM CDCA (grey bars) or DMSO as control (white bars). Cells were co-incubated with DMSO as control or 10 µM AG1478, 10 µM UO126, and 50 µM PD98059, respectively. DNA incorporation was determined after 7 days of culture (**A**). Following a total culture period of 14 days, DNA fluorescence as a readout of total cell number was quantified (**B**). To test functional consequence of cell accumulation, excess collagen deposition after 14 days of culture was determined (**C**). Results are shown as mean ± standard deviation (*n* = 6–9, * *p* < 0.05 CDCA vs. DMSO, ANOVA, post-hoc LSD).

Various inhibitor studies were performed to delineate whether activation of the MEK/Erk signaling pathway by bile salts may mediate their pro-proliferative effects in HSC. To block the EGFR/MEK/Erk signaling cascade on different levels, the following substances were used: (i) PD98059, an inhibitor of Erk activation; (ii) UO126, known to block upstream activation of MEK1/2; and (iii) AG1478, preventing upstream signaling by EGFR. While CDCA stimulation again resulted in significant increase in HSC proliferation (Figure 6A), cell mass (Figure 6B), and consequently collagen deposition (Figure 6C), inhibition of EGRF, MEK, or Erk by above-mentioned inhibitors abolished the pro-fibrotic effects of CDCA in HSC.

This suggests that EGFR/MEK-dependent signaling might be involved in mediating the direct pro-fibrotic effects of hydrophobic bile salts in HSCs, but further confirmation is needed.

## 4. Discussion

While bile salts accumulating in chronic cholestatic liver disease have been believed to promote liver fibrosis for decades, supporting in vivo evidence for this concept is lacking. Experimental evidence has been hampered by the fact that in all established animal models of cholestatic liver fibrosis, both liver fibrosis and cholestasis are secondary to biliary damage, which provokes strong pro-fibrotic signals in its own right. To overcome this limitation, we used the *Atp8b1^G308V/G308V^* mouse, which is a unique inducible model of hepatocellular cholestasis, independent of a biliary insult [27,28,34,35].

Chronic cholestasis was induced in these mice by CA feeding, in line with earlier studies (Figure 1). By supplementation of the diet with the predominant human hydrophobic bile salt GCDCA, enrichment of GCDCA in serum, liver, and bile could be achieved in the mice (Figure S3). Thus, a humanization of the bile salt pool was possible, shifting the composition of bile salts in mice from hydrophilic to hydrophobic bile salts. Chronic cholestasis in presence of GCDCA evoked pro-fibrotic signaling at the mRNA level (Figure 2), resulting in excess deposition of collagen in liver tissue (Figure 3). Notably, liver fibrosis only developed in cholestatic animals upon GCDCA supplementation. Cholestasis alone, in the context of the unaltered, i.e., more hydrophilic rodent bile salt pool, was insufficient to induce liver fibrosis (Figure S1). Furthermore, GCDCA supplementation alone, in absence of cholestasis, did not lead to liver fibrosis (Figure 3, wild-type mice). It has previously been speculated that rodents may be less prone to chronic, especially cholestatic liver disease due to their hydrophilic and presumably less toxic bile salt pool. Our data support this assumption and, importantly, are in line with our previous findings showing that a humanized bile salt pool, then induced by lack of hepatic rehydroxylation capacity due to knockout of cytochrome P450 (POR) [34], aggravated liver disease in *Mdr2^−/−^* mice.

We did not find evidence for proinflammatory cytokine expression or recruitment of inflammatory cells, which have been previously reported in animal models of biliary damage [38,39], but these mechanisms might also play a role in hepatocellular cholestasis.

Mechanisms by which cholestasis might promote liver fibrosis have largely remained elusive. Here, we demonstrate in vitro that hydrophobic, but not hydrophilic, bile salts promote proliferation and accumulation of HSCs, accompanied by excess deposition of collagen (Figure 4). This was associated with activation of the MEK/Erk signaling pathway (Figure 5). In the presence of inhibitors of this signaling cascade, pro-proliferative and pro-fibrotic actions of CDCA in HSC were abolished (Figure 6).

Previous studies have identified activation of pro-proliferative signaling pathways by bile salts in rat HSCs [40,41] and in the HSC cell line LX2 [42]. Our study demonstrated for the first time the functional consequences of this pro-proliferative signaling, namely, an increase in HSC mass and collagen deposition upon bile salt stimulation. While both hydrophilic and hydrophobic bile salts seemed to evoke pro-proliferative signaling in one previous study [40], our results were in line with more recent work supporting a predominant effect of hydrophobic bile salts [41]. The bile-salt-induced activation of EGFR/MEK/Erk signaling that we found in HSCs is in line with previous studies [40,41] and has been firmly established in hepatocytes in the past [12,43], indicative of a cell-type-independent mechanism of action. Thus, our results are in line with previous studies on the subject. Moreover, they add information on the functional consequences of bile-salt-induced pro-proliferative signaling in HSCs, namely cell mass expansion and collagen deposition.

In our study, conjugates of CDCA stimulated accumulation of HSCs to a lesser extent compared to CDCA (Figure S4B). This may have been a consequence of the specific chemical properties of GCDCA and TCDCA, which are fully ionized at physiologic pH and therefore prevented from passively entering into cells by diffusion. This could be predicted from their lower pK_a_ compared to CDCA [44], and is of physicochemical relevance. As we have previously demonstrated in human cholangiocytes in vitro, bile-salt-induced signaling can be determined by pK_a_ and extracellular pH [45,46]. It is important to note that HSCs in vivo reside in the space of Disse, which serves as a proton diffusion barrier and is acidic in pH (pH < 7.0) [47]. Thus, penetration of especially GCDCA may be underestimated in vitro under standard culture conditions. Further investigations into the influence of extracellular pH in HSC culture should therefore be the subject of future research.

Unconjugated bile salts are enriched in portal blood [44], and unconjugated CDCA in portal blood accounts for up to 40% [48], while in the systemic circulation, CDCA is mainly found in its conjugated forms, GCDCA and TCDCA. Thus, the mechanisms identified in this in vitro study may be of pathophysiological relevance in chronic cholestatic diseases, where CDCA and its conjugates are the most abundant, accumulating hydrophobic bile salts. Our in vitro results raise the question of whether feeding of unconjugated CDCA instead of GCDCA would suffice to induce a fibrotic phenotype in vivo. This will be explored in future studies.

Induction of cholestasis in *Atp8b1^G308V/G308V^* mice has been extensively studied, e.g., to unravel the mechanisms leading to cholestasis in patients with PFIC1 [27,28,35]. However, thus far, long-term cholestasis in the absence of liver damage, and its very own role in the pathogenesis of liver fibrosis, has not yet been investigated in this model. Therefore, our study is an important proof of principle study into the genesis of cholestatic liver fibrosis beyond PFIC1.

Our results may set the stage for the exploration of bile-salt-pool-modifying agents as anti-fibrotic treatments in cholestatic disease. It has been previously shown that inhibitors of the ileal bile salt uptake transporter ASBT improve liver damage in *Mdr2^−/−^* mice [49,50]. Our data support the view that changes in the composition of the bile salt pool may be the key mechanism of action, and therefore support the ongoing clinical exploration of such agents [51].

The fibrotic phenotype of our cholestatic model was rather modest. This reflects the often slowly progressive nature of chronic cholestatic diseases in humans. In most cholestatic disorders in humans, however, additional “hits” influence disease progression and may be important drivers of fibrotic remodeling. In PSC, for example, biliary inflammation may be an important cause for fibrotic remodeling, while secondary cholestasis may perpetuate disease progression. The modest phenotype in our models may thus reflect the situation for the majority of patients with chronic cholestatic liver disease. However, the modest effect size hampers the applicability of the model for studying involved pathways in vivo. It has been repeatedly demonstrated that genetic background has a great influence on the extent of fibrosis seen in cholestatic models, e.g., in the *Mdr2^−/−^* mouse, where BALBc seems to be the most susceptible background [52]. Therefore, translation of our model into different genetic backgrounds may produce a more robust phenotype, allowing the further study of involved pathways and therapeutic interventions.

## 5. Conclusions

For the first time, our study provides in vivo evidence for the generally accepted concept that human hydrophobic bile salts accumulating in chronic cholestatic liver disease may promote liver fibrosis. Our experiments emphasize the importance of the composition of the bile salt pool in cholestatic disease, indicating that the human hydrophobic bile salt GCDCA predisposes to liver fibrosis. One potential, hitherto under-recognized mechanism may be the direct pro-proliferative effects of hydrophobic bile salts in HSCs.

## Supplementary Materials

The supplementary materials are available online at https://www.mdpi.com/2073-4409/9/2/281/s1.
