# Supplementary material for: Glycochenodeoxycholate Promotes Liver Fibrosis in Mice with Hepatocellular Cholestasis
Source: Cells. 2020 Jan 23;9(2):281. doi: 10.3390/cells9020281 (PMC7072501; doi:10.3390/cells9020281)
Supplement: Supplementary file 1 [file cells-09-00281-s001.pdf]

Supplementary Figure 1

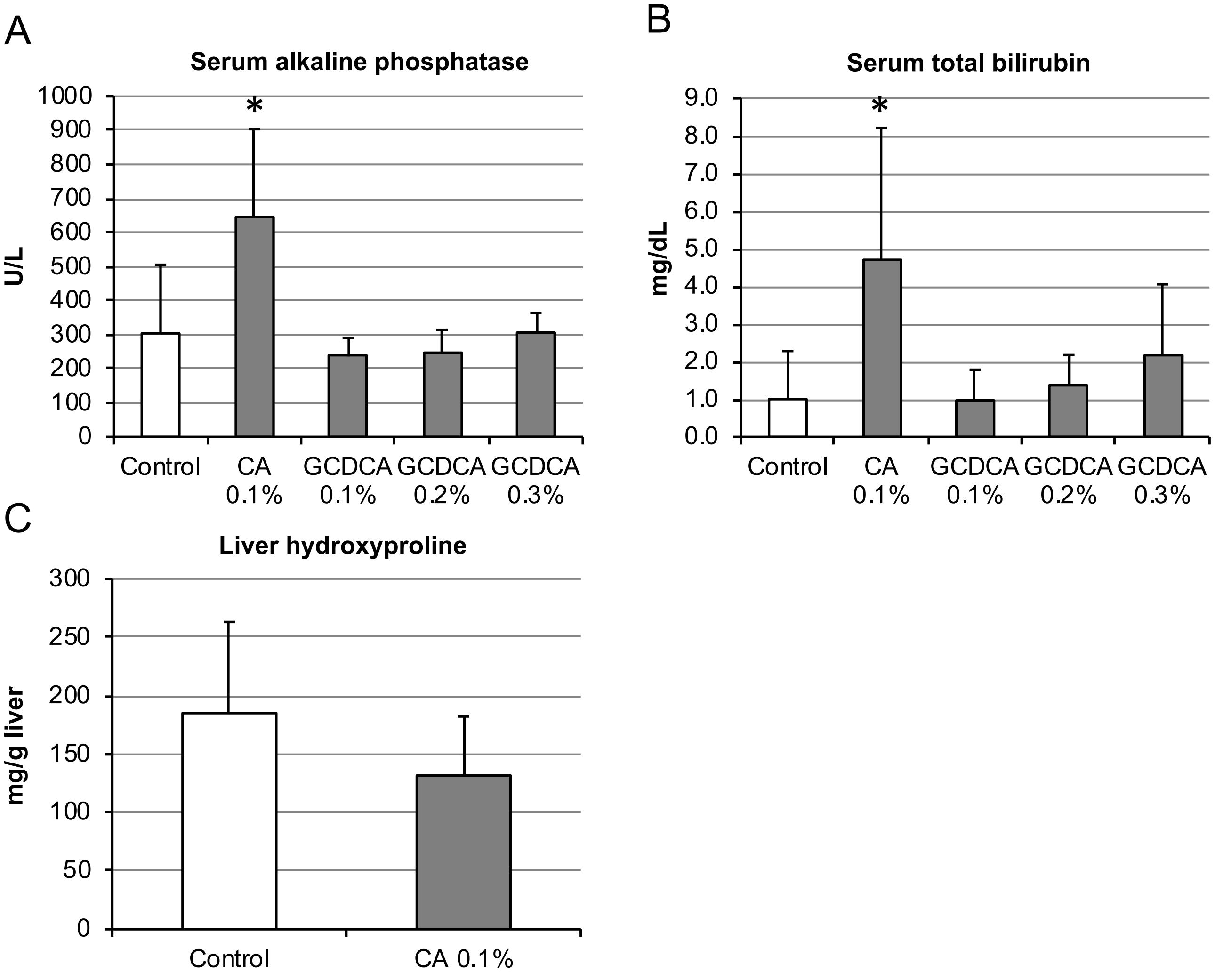

Supplementary Figure 1: CA feeding, but not GCDCA feeding, induces chronic cholestasis in *Atp8b1*$^{G308V/G308V}$ mice.

Atp8b1$^{G308V/G308V}$ mice were fed a standard diet (white bars) or a diet enriched with the indicated bile salts (w/w in diet, grey bars). Cholestasis was determined by measurement of serum values for alkaline phosphatase (A) and total bilirubin (B). Liver hydroxyproline was quantified to determine liver fibrosis (C). Results are shown as mean ± standard deviation (n=19 for Control, n=8 for CA 0.1%, n=9-12 for GCDCA-containing diets, *p<0.05, ANOVA, post-hoc LSD).

Supplementary Figure 2

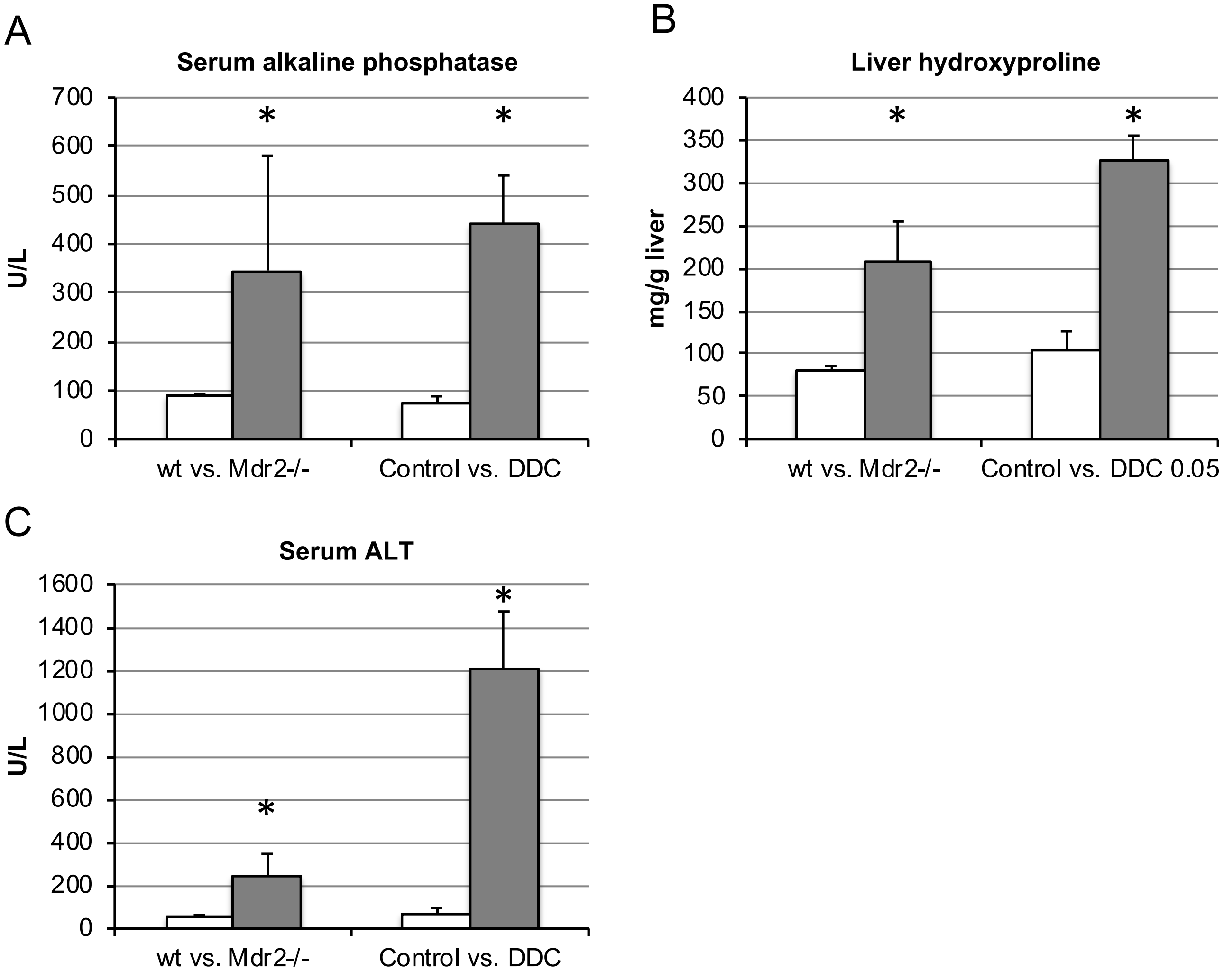

Supplementary Figure 2: Liver fibrosis in established models of cholestasis is associated with marked liver damage.

Key characteristics of established models of liver fibrosis associated with cholestasis were investigated for comparison with the phenotype of *Atp8b1*$^{G308V/G308V}$ mice. Wildtype FVB (white bar) and *Mdr2*$^{-/-}$ (grey bar) were sacrificed at 12 weeks of age. 8 weeks old C57/BL6 mice were fed a control diet (white bar) or DDC containing diet (0.05%, grey bar) for 8 weeks. Serum values for alkaline phosphatase (A) indicate cholestasis in these animals and liver hydroxyproline content (B) confirms development of liver fibrosis. Both models are associated with serious liver damage, as indicated by markedly increased serum activities of ALT. Results are shown as mean ± standard deviation (n=4-5, *p<0.05 wt vs. Mdr2 and control vs. DDC, respectively, t-test).

Supplementary Figure 3

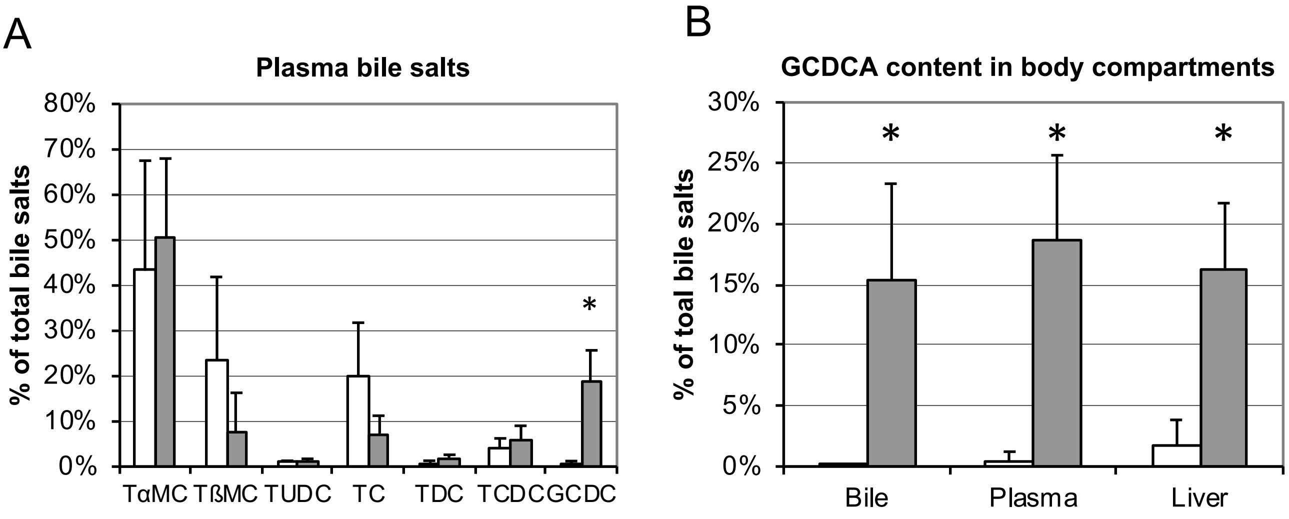

Supplementary Figure 3: GCDCA feeding induces a humanized bile salt pool in C57/BL6 mice.
Mice have a very pronounced rehydroxylation capacity for bile salts. We therefore tested, whether GCDCA supplementation with the diet leads to an enrichment in different body compartments or is metabolized to beta-muricholic acid (βMCA).
Mice were fed a purified semisynthetic diet (K4068.02; Arie Blok Diervoeders, Woerden, The Netherlands) either or not supplemented with GCDCA (0.1% w/w) for 4 weeks and steady state composition of the bile salt pool was analyzed by reverse-phase high-performance liquid chromatography (HPLC). White bars represent animals fed a control diet, grey bars represent GCDCA fed animals. Results are shown as mean ± standard deviation of all detectable bile salts (n=4, each, *p<0.05 control vs. GCDCA, t-test) and reflect plasma bile salt composition (A) and relative GCDCA content in indicated body compartments (B).

Supplementary Figure 4

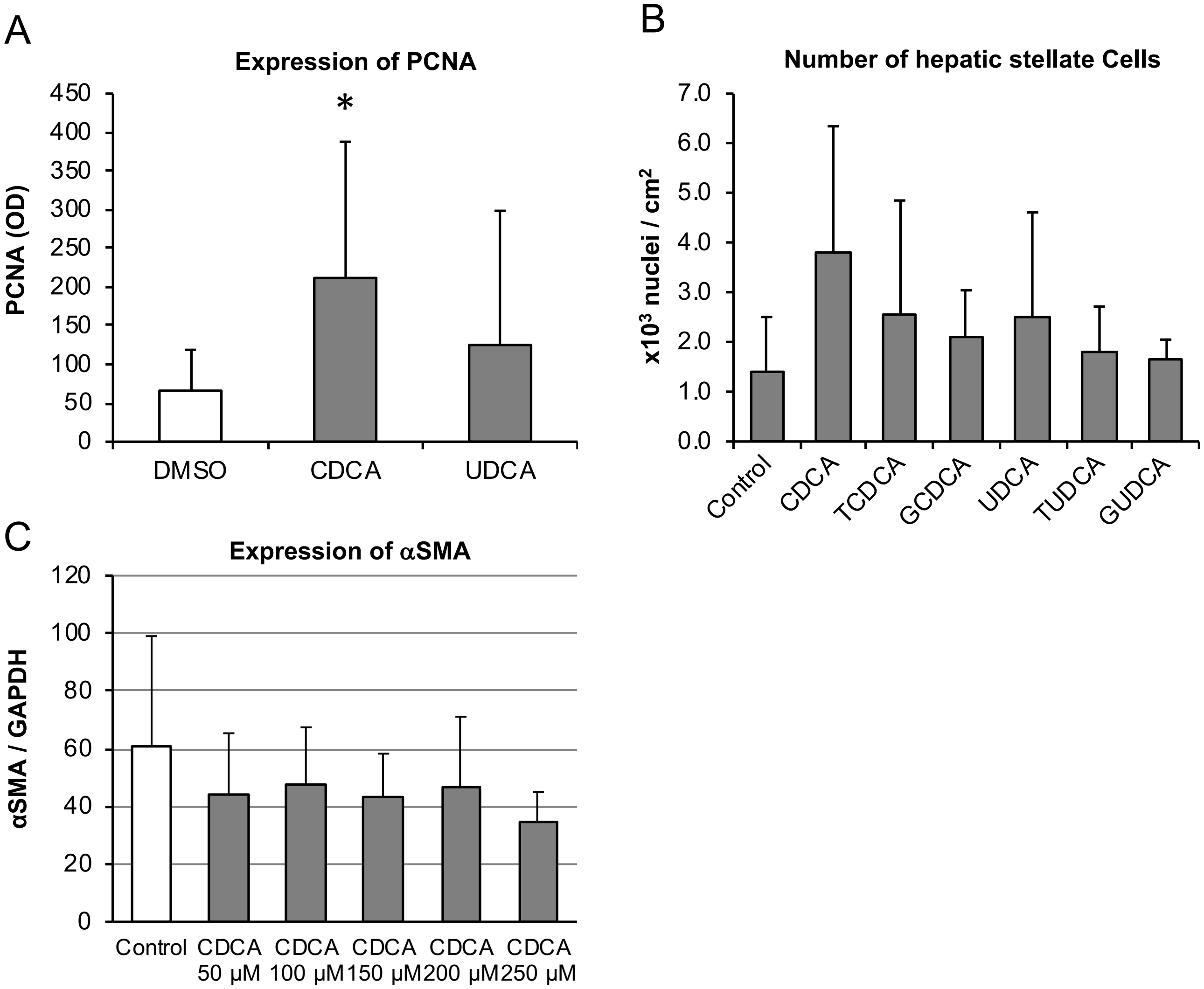

Supplementary Figure 4: Hydrophilic and conjugated bile salts are less potent in inducing proliferation / accumulation of hepatic stellate cells (HSC).
HSC were isolated from wildtype mice as described and stimulated with indicated bile salts. (A) After 4 days of stimulation with CDCA or UDCA at 100uM, PCNA expression was determined by Western blotting (n=6-8). (B) Following long term stimulation of HSC for 14 days at 250uM, absolute numbers of cells were quantified (n=4-6). (C) Hepatic stellate cells were stimulated with CDCA at the indicated concentrations for 14 days and expression of αSMA was determined relative to the housekeeping protein GAPDH by Western Blotting. Results are given as mean ± standard deviation (*$p<0.01$, ANOVA, post-hoc LSD (A), Tukey test (B+C)).
